# Alternative or complementary *attitudes* toward alternative and complementary *medicines*

**DOI:** 10.1186/s12906-019-2490-z

**Published:** 2019-04-08

**Authors:** Fabrice Berna, Anja S. Göritz, Amaury Mengin, Renaud Evrard, Jacques Kopferschmitt, Steffen Moritz

**Affiliations:** 1 Hôpitaux Universitaires de Strasbourg, 1 place de l’Hôpital, Clinique Psychiatrique, F-67091 Strasbourg Cedex, France; 2 Université de Strasbourg, Fédération de Médecine Translationnelle de Strasbourg, Strasbourg, France; 3 Inserm U1114, Strasbourg France ; Fondation FondaMental, Créteil, France; 4 CUMIC, Collège Universitaire des Médecines Intégratives et Complémentaires, Nantes, France; 5 Occupational and Consumer Psychology, Freiburg University, Engelbergerstraße 41, D-79085 Freiburg, Germany; 6 INTERPSY (EA 4432), Université de Lorraine, 23 Boulevard Albert 1er, F-54000 Nancy, France; 7 University Medical Center Hamburg-Eppendorf, Department of Psychiatry and Psychotherapy, Martinistr. 52, D-20246 Hamburg, Germany

**Keywords:** Complementary and alternative medicine, Treatment preference, Attitudes, Severe illness

## Abstract

**Background:**

Integrative and complementary health approaches (ICHA) are often pursued by patients facing chronic illnesses. Most of the studies that investigated the factors associated with ICHA consumption have considered that the propensity to use ICHA is a stable or fixed characteristic of an individual. However, people may prefer using ICHA in some situations and not in others, depending on the characteristics of the illness to face. Moreover, the attitude toward ICHA may differ within a single individual and between individuals so that ICHA can be used either in addition to (i.e., complementary attitude) or in place of (i.e., alternative attitude). The present study aimed at examining distinct patterns of attitudes toward ICHA in people hypothetically facing chronic illnesses that differed according to severity and clinical expression.

**Methods:**

We conducted a web-based study including 1807 participants who were asked to imagine that they had a particular chronic illness based on clinical vignettes (mental illnesses: depression, schizophrenia; somatic illnesses: rheumatoid arthritis, multiple sclerosis). Participants were invited to rate their perceived distress and social stigma associated with each illness as well as its perceived treatability. They also rated their belief in treatment effectiveness, and their treatment preference. Four patterns of treatment choice were determined: strictly conventional, weak or strong complementary, and alternative. Bayesian methods were used for statistical analyses.

**Results:**

ICHA were selected as complementary treatment option by more than 95% of people who hypothetically faced chronic illness. The complementary attitude towards ICHA (in addition to conventional treatment) was more frequent than the alternative one (in place of conventional treatment). Factors driving this preference included employment status, severity of illness, age and perceived distress, social stigma and treatability of the illness. When the label of illnesses was included in the vignettes, patterns of treatment preference were altered.

**Conclusions:**

This study provides evidence that “medical pluralism” (i.e., the integration of ICHA with conventional treatment) is likely the norm for people facing both mental or somatic illness. However, our result must be interpreted with caution due to the virtual nature of this study. We suggest that taking attitudes toward ICHA into account is crucial for a better understanding of patients’ motivation to use ICHA.

**Electronic supplementary material:**

The online version of this article (10.1186/s12906-019-2490-z) contains supplementary material, which is available to authorized users.

## Background

Integrative and complementary health approaches (ICHA) is the current denomination of the US National Institutes of Health [1] for “medicines” that were previously labeled as soft, parallel, or complementary and alternative (CAM). While the use of ICHA for minor daily hassles in healthy people is not of major concern, ICHA are also and frequently used by patients with severe physical or mental illness [2–5]. Moreover, ICHA is often the first treatment option chosen by patients before seeking help with conventional medicine [6–8]. This attitude is observed despite controversies about the efficacy of ICHA and the fact that at least some of them are not derived from evidence-based medicine and/or not research with rigorous research designs (e.g., [9]), thereby raising the question of the factors that drive patients’ propensity to consider ICHA as valuable treatment option.

The label “alternative medicine” has been historically removed considering that non-conventional medicines usually represent rather complementary than alternative options to conventional treatments. This goes in hand with the development of the “medical pluralism” (i.e., the use of multiple forms of healthcare [8, 10]) that has dramatically increased in most industrialized countries [11–13] but also in low and middle income countries [14–17]. However, independent of the kind of medicine, ICHA are used by consumers sometimes in addition to, sometimes in place of conventional treatments. This emphasizes the need to distinguish ICHA on the one side from the way it is used on the other; the latter probably depending on the attitudes (complementary vs. alternative) of the consumer toward ICHA.

Most of the studies that have investigated the factors associated with ICHA use have investigated people disclaiming ICHA use (in some cases, people facing particular illnesses) and examined the socio-demographic factors associated with ICHA use [18, 19]. By doing so, those studies have implicitly considered that the propensity to use ICHA is a stable or fixed characteristic of an individual. However, ICHA use could vary according to the kind of illness faced by the person or by the severity of this illness, so that people may prefer ICHA in some situations and not in others. Similarly, the same individual could use ICHA in addition to conventional treatment when facing a particular illness (i.e., complementary attitude) but refuse conventional treatment for another illness and use only ICHA (i.e., alternative attitude).

The aim of the present study was to examine distinct patterns of attitudes toward ICHA in a large sample of participants. In this study, complementary and alternative attitudes toward ICHA were examined in people hypothetically facing chronic illnesses that differed according to their severity and clinical expression. We decided to contrast chronic mental vs. somatic illnesses to examine whether ICHA are more easily chosen to treat mental compared to somatic illnesses, this reflecting societal bias of mental illness perceived as less biologically founded and thus less treatable with conventional medicine [2].

## Methods

The present online study recruited German-speaking participants via the participant pool WiSoPanel [20, 21] (http://www.wisopanel.net). The link to the study was sent to 12,134 people, and responses were collected within a week (see previous publication using the same study design [22]). The likelihood of bias was reduced by the use of diverse channels and sources for the recruitment of the participant pool, whose demographic characteristics resemble the general population. People had registered to be invited to participate in online studies of all kinds and topics. Thus, it was unlikely that a selection bias occurred with regard to an affinity to the study topic. Moreover, all eligible members of the pool received the invitation to the study at hand. Finally, this study is based on a census, not a sample of the participant pool, this reducing notably the risk of self-selection bias on the level of this individual study.

The study was conducted as part of a research grant awarded by the German Research Foundation (DFG; www.dfg.de) to ASG (grant identifier: GO 1107/4–1). The DFG’s board of ethics passed the research proposal that underlies the present study. Additional approval by other ethics committees (e.g., from universities) are not required by projects funded by DFG. After reading the short description of the purpose of the study (see Section 2.2.) all participants gave their written informed consent on line, in accordance with DFG’s board of ethics. The investigation was carried out in accordance with the latest version of the Declaration of Helsinki [23].

### Study design

The questionnaire used in this study was the same as that used in a previous publication [22]. For each participant, the design of the study included a comparison between two illnesses: one mental and one somatic. Four levels of severity were used for each illness, so that eight clinical vignettes were assessed by each participant.

Mental illnesses included schizophrenia (SZ) and recurrent depression (RD), and somatic illnesses included rheumatoid arthritis (RA) and multiple sclerosis (MS). All four illnesses have in common a poor prognosis, an elevated relapse rate, a need for life-long medication.

Eight sets of combinations (A1 to A4 and B1 to B4) of mental and somatic illnesses were created in order to compare all pairs of somatic and psychiatric illness (see description in the Additional file 1). Participants who entered the study were randomly assigned to one out of eight combinations. In combinations B1 to B4, we included the name of the illness in the last two presented vignettes.

### Clinical vignettes of illnesses

At the start of the study, participants received the following instruction: “In the present study we are interested in investigating how people would behave when facing chronic illnesses. In the following pages, 8 short clinical vignettes of chronic illnesses will be presented to you. For each of them, you will be asked to imagine that you would personally suffer from this particular illness and then to assess how much of a burden this would be to you and which treatment option you would prefer.”

Then, the following sentence was presented at the start of each clinical vignette: “Imagine you suffer from a chronic illness that presents itself with the following symptoms [*specific symptoms*]”. The respective specific symptoms of the illness (somatic or mental) were then entered into the blanks (see Table 1); we checked the validity of each illness vignette with help of specialists of the illness. Given that symptoms description may activate different social representations of illnesses severity, the severity of illnesses vignettes was controlled in creating four levels of illness severity. Basically, the frequency of relapse (high or low) and the severity of symptoms during acute episodes (high or low) were both described in the next two sentences of the vignettes. We obtained therefore four levels of illness severity by combining the frequency of relapse and the symptoms severity (see Additional file 1).

**Table 1:** Description of the chronic illnesses (SZ: schizophrenia, RD: recurrent depression, MS: multiple sclerosis, RA: rheumatoid arthritis)

Type of illness	Mental	SZ	“You have the feeling that your environment is hostile or threatening. You also have frequent odd perceptions, for instance hearing voices, despite that nobody is speaking around you. You also suffer from lack of motivation and tend to neglect your hygiene. This is noticed by other people rather than by yourself.”
		RD	“You experience a groundless sadness and lose your joy of life. You also notice that you lack motivation or inner impulse for your daily life activities. Your capacities to concentrate are considerably reduced (for instance when reading or watching TV)”
	Somatic	MS	“You suffer from a lack of (muscular) strength in your legs. This weakness waxes and wanes from day to day and is unpredictable. You also have deficits in sensitivity in particular parts of your body and you suffer from enduring impaired vision.”
		RA	“You suffer from pain in yours joints, particularly in your knees, elbows, hands and fingers, which you feel more or less constantly. These pains are disturbing in your daily life and also at night.”
Level of severity	Frequency of relapse	High	“The illness evolves with frequent episodes that occur several times per year. It has a poor prognosis and worsens if you do not adhere to your treatment appropriately.”
		Low	“The illness evolves with episodes that occur infrequently (one episode every 3 to 5 years). the illness may nevertheless worsen and have a poor prognosis, particularly if you do not adhere to your treatment appropriately.”
	Severity of symptoms during episodes	High	“During acute episodes, you must stay at home because of your symptoms. Also, you often have to be hospitalized for several weeks because of the severity of the symptoms.”
		Low	“During acute episodes, you can keep dealing with your daily life activities, for instance going at work. Nevertheless, you feel that your functioning is significantly impaired.”
Example of the clinical description of recurrent depression with low frequency of relapse and high severity of symptoms during episode, without the label of illness: “Imagine you suffer from a chronic illness that presents itself with the following symptoms: You experience a groundless sadness and lose your joy of life. You also notice that you lack motivation or inner impulse for your daily life activities. Your capacities to concentrate are considerably reduced (for instance when reading or watching TV). The illness evolves with episodes that occur infrequently (one episode every 3 to 5 years). the illness may nevertheless worsen and have a poor prognosis, particularly if you do not adhere to your treatment appropriately During acute episodes, you must stay at home because of your symptoms. Also, you often have to be hospitalized for several weeks because of the severity of the symptoms.” Example of the clinical description of recurrent depression with low frequency of relapse and high severity of symptoms during episode and that includes the label of illness: “Imagine you suffer from recurrent depression that presents itself with a groundless sadness and a loss of your joy of life. You also notice that you lack motivation or inner impulse for your daily life activities. Your capacities to concentrate are considerably reduced (for instance when reading or watching TV). The illness evolves with episodes that occur infrequently (one episode every 3 to 5 years). the illness may nevertheless worsen and have a poor prognosis, particularly if you do not adhere to your treatment appropriately During acute episodes, you must stay at home because of your symptoms. Also, you often have to be hospitalized for several weeks because of the severity of the symptoms.” NB: The illness description associating both low frequency of relapse and low symptoms severity was replaced in Conditions B1-B4 by the description of illness including its name, high frequency of relapse and high symptoms severity.			

We randomized the presentation order of the eight clinical vignettes.

### Ratings of distress, treatability and perceived social stigma associated with chronic illness

**Fig. 1:**
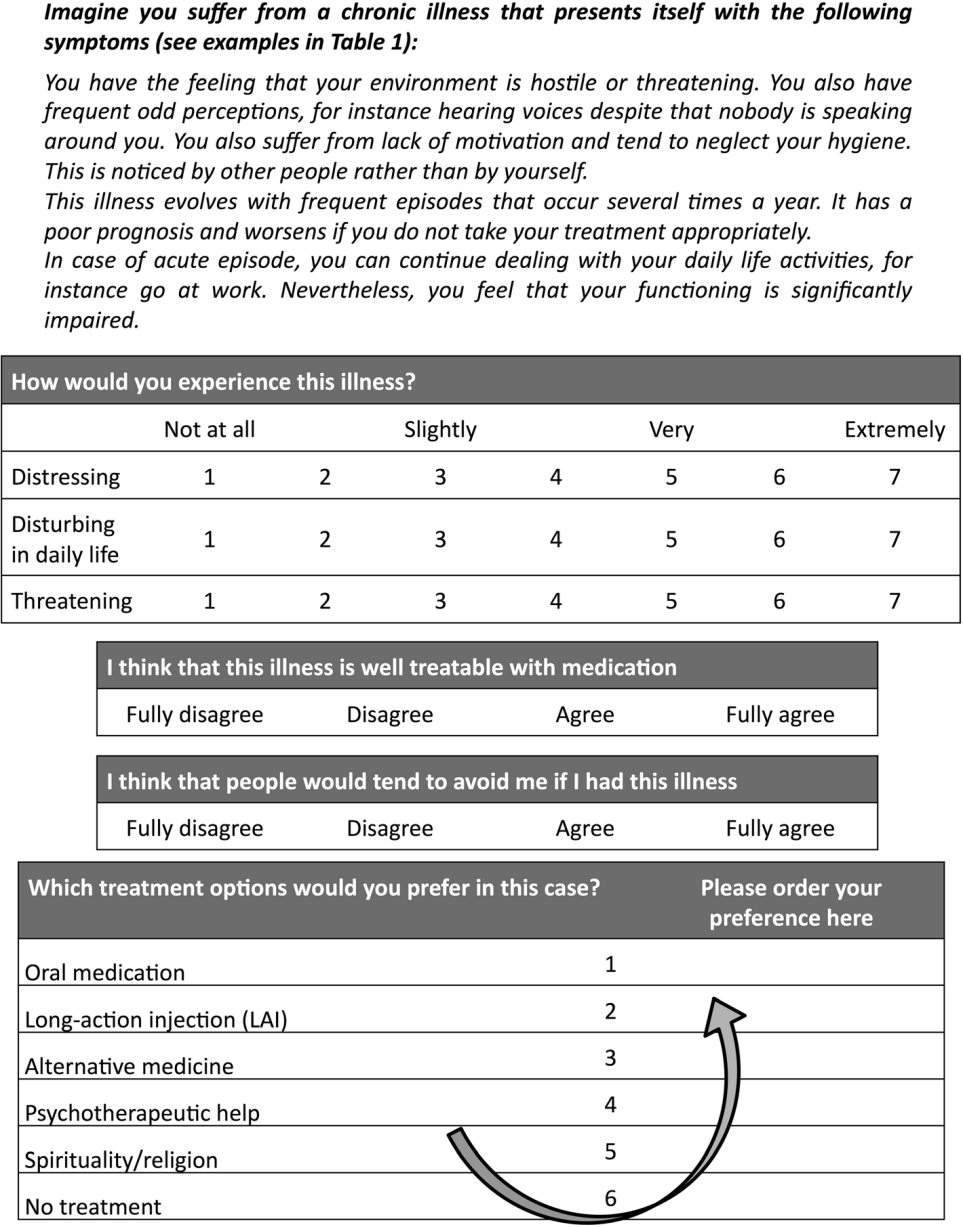
Description of the study protocol

After each vignette, participants were invited to rate on 7-point Likert scales how they would experience the illness in terms of subjective burden, daily life impairment, feeling of threat (see Fig. 1). We calculated a composite distress score using the mean of the three scales.

Next, they answered two questions using four-point Likert scales (1 = fully disagree, 2 = rather disagree, 3 = rather agree, 4 = fully agree): “I think that this illness is well treatable with medication” (treatability score) and “I think that people would tend to avoid me if I had this illness” (perceived social stigma score).

### Treatment preference

Next, participants ranked the treatment options according to personal preference among the following: 1) oral medication, 2) long-acting injection (LAI) of the medication, 3) alternative medicine (e.g., acupuncture, homeopathy, or other), 4) psychotherapeutic help (psychological or psychiatric), 5) spirituality/religion or, 6) no treatment. These options were presented in a list on the left side of the screen. Participants had to drag and drop the treatments one after another into a box on the right side. They were asked to order them from the most to the least preferred treatment option (see Fig. 1). We randomized the presentation order of treatment options in the left box. Participants were free to select as much treatment options as needed.

### Belief in the effectiveness of treatment options

Next, we invited participants to complete other questions to assess their belief in the effectiveness of each of the treatment options presented above for both chronic illness. Then, for each illness, participants were invited to rate on a 4-point scale (1 = fully disagree, 2 = rather disagree, 3 = rather agree, 4 = fully agree) the following statement: “I consider the following treatment option as efficient for schizophrenia (or RD, MS, RA)” followed by each treatment option. Hence, a score of belief in treatment effectiveness was obtained for the somatic and mental illnesses for each participant.

#### Statistical analyses

For each clinical vignette, treatment choices were categorized in four patterns of choice (see concrete examples into additional file 2):

1. “Strictly conventional” for people selecting pill, LAI or psychotherapy as unique treatment option excluding ICHA or spiritual guidance
2. “Weak complementary” for people selecting pill or LAI as first treatment option combined with ICHA as further treatment option
3. “Strong complementary” for people selecting ICHA or spiritual guidance as first treatment option combined with conventional treatment as further treatment option
4. “Alternative” for people selecting ICHA or spiritual guidance as unique treatment option, excluding conventional treatment options (pill, LAI or psychotherapy)

Statistical analyses were performed using Bayesian methods (rjags [25] for R software [26]). Sociodemographic and cognitive variables were compared between groups using univariate analyses. The pattern of choice for each vignette was analyzed with multilevel multinomial models using choice pattern as Level 1 and subject as Level 2; the weak complementary option was entered as the reference category. Predictor variables included linear variables (distress, treatability, and perceived social stigma) and categorical variables: gender, level of schooling (4 categories), employment status (5 categories), type of illness (mental vs. somatic), frequency of relapse (low vs. high), intensity of symptoms (low vs. high). To investigate whether naming the illness influenced treatment preference, our comparisons were restricted to the vignettes associated with the highest level of illness severity (i.e., high severity of symptoms and high frequency of relapse). Type of illness (mental vs. somatic) and name of illness (present vs. absent) were used as predictor variables. The influence of each predictor was examined first in separate univariate analyses. Multivariate analyses including all relevant predictors was finally conducted.

Analyses were performed using non-informative priors for both the univariate and multivariate analyses (normal distribution N [mean +/− standard deviation] for the log-OR = N [0; 0.04]), that amounts to expect an odds ratio (OR) equal to 1 (for categorical predictors) with a 95% credible interval (CI) of 0.05 to 20 (see JAGS script in additional file 3).

## Results and analyses

### Participants

The study included 1938 participants (16%); this rate is similar to other online studies from WiSoPanel [24]. They were assigned randomly to one of eight conditions contrasting the different vignettes. We arbitrary decided to exclude participants aged above 75 years (*n* = 40) in order to avoid issues related to advanced age. We also excluded participants who completed the study too fast (i.e. a time duration below percentile 5; *n* = 71) in order to avoid possible non-reliable responses relating to speeding at study completion. Finally, we excluded people disclosing at the end of the study that they had not responded sincerely (*n* = 30).

Statistical analyses were therefore performed on 1807 individuals. Each of the eight conditions included a mean of 226 participants (range = 213 to 241). Conditions did not differ regarding age, gender and level of education. Participants’ mean age ranged between 46.6 and 49.4 years (range 19 to 75) and each condition comprised 57.7% up to 61.9% of women.

### Results

#### Descriptive results

First, 64.5% of the participants selected treatment options that fell in the same pattern of choice whatever the four vignettes of mental illnesses. This proportion was similar (62.9%) for somatic illnesses. In contrast, only 46.8% selected the same pattern of choice across the eight vignettes: This indicates that response patterns differed between somatic and mental illnesses in 16.0 to 17.7% of participants (see Table 2).

**Table 2:** Diversity of treatment options selected by the participants by type of illness

Number of distinct treatment options	All illnesses		Mental Illnesses		Somatic illnesses	
	*N*	(%)	*N*	(%)	*N*	(%)
1 option	421	(46.8)	580	(64.5)	565	(62.9)
2 options	396	(44.1)	282	(31.4)	311	(34.6)
3 options	78	(8.7)	35	(3.9)	23	(2.6)
4 options	4	(0.4)	2	(0.2)	0	(0.0)
Total	899	(100.0)	899	(100.0)	899	(100.0)

People selecting the same option belonged to the weak complementary pattern in 69.3 to 75.9% of cases. The strictly conventional and alternative options represented less than 11.2 and 5.2% of the single selected options. When a combination of options was selected, the most frequent one was weak plus strong complementary (68.8 to 73.5%). Combinations including the strictly conventional option represented 18.2 to 20.3% of all combinations and those including the alternative option 20.1 to 25.9% (see Table 3).

**Table 3:** Details of the treatment options selected by the participants by type of illness

Treatment options selected in participants with	All illnesses		Mental illnesses		Somatic illnesses	
	*N*	(%)	*N*	(%)	*N*	(%)
Single choice options						
1- Strictly conventional	43	(10.2)	65	(11.2)	49	(8.7)
2- Weak complementary	319	(75.8)	402	(69.3)	408	(72.1)
3- Strong complementary	50	(11.9)	83	(14.3)	93	(16.6)
4- Alternative	9	(2.1)	30	(5.2)	15	(2.7)
Total	421	(100.0)	580	(100.0)	565	(100.0)
Two choice options						
1 + 2	51	(12.9)	37	(13.1)	44	(14.2)
1 + 3	1	(0.3)	1	(0.4)	3	(1.0)
1 + 4	6	(1.5)	5	(1.8)	7	(2.3)
2 + 3	291	(73.5)	200	(70.9)	214	(68.8)
2 + 4	33	(8.3)	21	(7.5)	27	(8.7)
3 + 4	14	(3.5)	18	(6.4)	16	(5.1)
Total	396	(100.0)	282	(100.0)	311	(100.0)
Three choice options						
1 + 2 + 3	11	(14.1)	5	(14.3)	6	(26.1)
1 + 2 + 4	22	(28.2)	7	(20.0)	5	(21.7)
1 + 3 + 4	2	(2.6)	1	(2.9)	0	(0.0)
2 + 3 + 4	43	(55.1)	22	(62.9)	12	(52.2)
Total	78	(100.0)	35	(100.0)	23	(100.0)
All combinations including						
Strictly conventional	97	(20.3)	58	(18.2)	65	(19.5)
Weak complementary	455	(95.2)	294	(92.2)	308	(92.2)
Strong complementary	366	(76.6)	249	(78.1)	251	(75.2)
Alternative	124	(25.9)	76	(23.8)	67	(20.1)

*Note*: *N* indicate the number of participants who selected the distinct presented options/combinations of treatment. This number refers to all clinical vignettes (column All illnesses), to mental illnesses only (column Mental illnesses) or to somatic illnesses only (column Somatic illnesses)

The lower part of the table indicates the total number of participants that selected combinations of treatment including one of the existing options (either strictly conventional, weak complementary, strong complementary and alternative) at least once

#### Univariate analyses

Regarding categorical variables, employment status altered pattern of choice whereas gender and level of schooling had no clear influence. Preference for the alternative over weak complementary option was rarer in unemployed participants compared to all other categories of participants. Preference for conventional over weak complementary option was more frequent in students compared to working participants and less frequent in students compared to unemployed participants (data not shown).

Participants clearly preferred strong complementary and alternative options (over the weak complementary option) for illnesses with low frequency of relapse or low intensity of symptoms. Participants preferred both strictly conventional and alternative options (over weak complementary option) when facing mental vs. somatic illnesses. They also preferred weak complementary over strong complementary when facing mental vs. somatic illnesses.

Regarding linear predictors, a clear influence of age, distress, treatability and social stigma was found. Age increased the preference for weak complementary over both alternative and strictly conventional options. Distress increased the preference for weak complementary over all other options. Treatability increased the preference for strictly conventional option and decreased that for alternative option. Social stigma increased the preference for strictly conventional option and decreased that for strong complementary and alternative options.

With regard to the belief in the efficacy of treatment, we aggregated beliefs for pill and LAI in a single score of belief in efficacy of conventional treatment. The same was performed for belief in the efficacy of complementary treatment (complementary + spiritual). None of the scores of beliefs in efficacy clearly influenced the preference for any treatment option.

These aforementioned results were obtained in the combinations A1 to A4; roughly similar results were found with linear and categorical predictors in the combinations B1 to B4 that explored the influence of naming the illness in the vignettes associated with the highest severity level. In addition, results showed that participants switched from the strong to the weak complementary option when the illness was named in the vignettes compared to the vignettes reporting the only symptoms.

The percentage of treatment options and rating scores associated with each treatment option are reported in Table 4.

**Table 4:** Percentage of treatment options and rating scores associated with each treatment option

Categorical variables (% per raws)		Strictly conventional (C)	Weak complementary (W)	Strong complementary (S)	Alternative
frequency of relapse	high	9.45	64.24	21.44	4.87
	low	9.40	60.96	23.30	6.34
intensity of symptoms	high	9.48	64.79	20.88	4.84
	low	9.37	60.40	23.86	6.37
type of illness	mental	10.23	61.54	21.58	6.65
	somatic	8.62	63.65	23.16	4.56
education level	< 9 years	10.61	62.21	23.26	3.92
	O level	8.44	62.42	24.51	4.63
	A level	9.28	61.50	22.17	7.06
	University and above	10.25	63.78	19.96	6.01
employment status	working	8.85	62.85	22.87	5.43
	pupil / student	12.90	54.26	23.94	8.91
	retired	9.40	65.27	18.69	6.64
	unemployed	5.77	71.39	21.63	1.20
	other	12.50	60.74	22.66	4.10
Linear variables		Strictly conventional (C)	Weak complementary (W)	Strong complementary (S)	Alternative
distress	*M*	5.19	5.50	5.18	4.39
(range 1–7)	*SD*	(1.30)	(1.25)	(1.36)	(1.49)
treatability	*M*	2.85	2.92	2.78	2.14
(range 1–5)	*SD*	(0.73)	(0.67)	(0.69)	(0.75)
social stigma	*M*	2.44	2.38	2.25	2.15
(range 1–5)	*SD*	(0.92)	(0.90)	(0.93)	(0.89)
belief in efficacy of	*M*	1.63	2.04	2.48	2.40
complementary treatments (range 1–5)	*SD*	(0.67)	(0.66)	(0.66)	(0.86)
belief in efficacy of	*M*	3.12	3.27	3.01	2.32
conventional treatments (range 1–5)	*SD*	(0.74)	(0.61)	(0.67)	(0.92)

#### Multivariate analyses

The variables entered in the model were frequency of relapse, intensity of symptoms, type of illness, employment status, age, distress, treatability and social stigma. Results indicated that influence of age, distress, treatability and social stigma remained unchanged. Regarding employment status, the preference for alternative option over weak complementary was still more rare in unemployed participants compared to other participants and more frequent in retired compared to working participants (data not shown). The preference for strictly conventional treatment over weak complementary was also less frequent in unemployed participants compared to studying, working and retired participants.

The frequency of relapse did no longer influence treatment preference, and strong complementary was preferred to weak complementary for illness with lower intensity of symptoms. Finally, the preference for both strictly conventional and alternative options compared to weak complementary options remained clear for mental vs. somatic illnesses.

In combinations B1 to B4, participants still switched from the strong to the weak complementary option if the illness was named in the vignettes and if all other clear factors were included in the multivariate model.

Results of both univariate and multivariate analyses are reported in Table 5.

**Table 5:** Univariate and multivariate analyses of the factors influencing preference for treatment

		Univariate analyses					Multivariate analyses				
Conditions A1 to A4				credible interval					credible interval		
categorical variables	Comparisons	*M*	*SD*	2.5%	97.5%	*Pr*(OR > 1)	*M*	*SD*	2.5%	97.5%	*Pr*(OR > 1)
frequency	W vs. C	0.992	0.086	0.837	1.175	0.461	0.944	0.083	0.795	1.122	0.254
(high = 1, low = 2)	W vs. S	**1.157**	**0.076**	1.017	1.315	0.987	1.088	0.071	0.957	1.236	0.901
	W vs. A	**1.352**	**0.147**	1.096	1.671	0.997	1.091	0.130	0.866	1.374	0.769
intensity of symptoms	W vs. C	0.985	0.086	0.830	1.166	0.429	0.917	0.082	0.770	1.091	0.164
(high = 1, low = 2)	W vs. S	**1.263**	**0.084**	1.108	1.437	> 0.999	**1.168**	**0.078**	1.026	1.330	0.990
	W vs. A	**1.368**	**0.149**	1.108	1.691	0.998	1.121	0.135	0.890	1.416	0.832
type of illness	W vs. C	**0.803**	**0.070**	0.678	0.952	0.006	**0.838**	**0.080**	0.696	1.008	0.031
(mental = 1, somatic = 2)	W vs. S	**1.132**	**0.075**	0.995	1.288	0.970	1.029	0.073	0.896	1.180	0.657
	W vs. A	**0.647**	**0.071**	0.523	0.801	< 0.001	**0.674**	**0.086**	0.527	0.863	0.001
linear variables	Comparisons	*M*	*SD*	2.5%	97.5%	*Pr*(Beta> 0)	*M*	*SD*	2.5%	97.5%	*Pr*(Beta> 0)
age	W vs. C	**−0.024**	**0.009**	−0.041	−0.006	0.003	**− 0.016**	**0.006**	− 0.029	− 0.003	0.007
	W vs. S	− 0.010	0.009	−0.027	0.007	0.129	0.002	0.006	−0.009	0.013	0.654
	W vs. A	**− 0.029**	**0.009**	− 0.047	− 0.011	0.001	**− 0.026**	**0.007**	− 0.041	− 0.012	< 0.001
distress	W vs. C	**−0.105**	**0.037**	−0.177	− 0.031	0.003	**−0.171**	**0.040**	−0.250	− 0.091	< 0.001
	W vs. S	**−0.111**	**0.031**	−0.172	−0.050	< 0.001	**−0.079**	**0.032**	−0.143	− 0.016	0.007
	W vs. A	**−0.571**	**0.044**	−0.658	−0.486	< 0.001	**−0.518**	**0.049**	−0.615	− 0.422	< 0.001
treatability	W vs. C	**0.147**	**0.071**	0.010	0.286	0.982	**0.138**	**0.069**	0.003	0.274	0.977
	W vs. S	−0.002	0.057	−0.114	0.111	0.488	−0.084	0.054	−0.191	0.022	0.060
	W vs. A	**−1.338**	**0.084**	−1.501	−1.177	< 0.001	**−1.266**	**0.082**	−1.427	−1.106	< 0.001
social stigma	W vs. C	**0.151**	**0.053**	0.049	0.254	0.998	**0.141**	**0.060**	0.023	0.260	0.991
	W vs. S	**−0.171**	**0.043**	−0.255	−0.087	< 0.001	**−0.137**	**0.047**	−0.230	−0.044	0.002
	W vs. A	**−0.268**	**0.064**	−0.394	−0.143	< 0.001	−0.106	0.079	−0.261	0.049	0.090
belief in efficacy	W vs. C	−0.033	0.083	−0.197	0.131	0.347					
of complementary	W vs. S	−0.019	0.073	−0.163	0.123	0.395					
treatments	W vs. A	0.104	0.094	−0.081	0.289	0.865					
belief in efficacy	W vs. C	−0.031	0.078	−0.182	0.121	0.344					
of conventional	W vs. S	0.052	0.066	−0.077	0.181	0.784					
treatments	W vs. A	−0.079	0.090	−0.254	0.098	0.189					
Conditions B1 to B4		Univariate analyses					Multivariate analyses				
categorical variables	Comparisons	*M*	*SD*	2.5%	97.5%	*Pr*(OR > 1)	*M*	*SD*	2.5%	97.5%	*Pr*(OR > 1)
name of illness	W vs. C	1.006	0.134	0.779	1.303	0.518	1.008	0.133	0.781	1.301	0.524
(absent = 1. present = 2)	W vs. S	**0.702**	**0.068**	0.582	0.847	< 0.001	**0.741**	**0.066**	0.623	0.881	< 0.001
	W vs. A	0.806	0.167	0.542	1.193	0.142	0.790	0.173	0.519	1.195	0.133

*Note*: Comparisons: W=Weak complementary (reference category), C = strictly Conventional, S=Strong complementary, A = Alternative

Results are presented as OR or Beta with a 95% CI, with the probability of the OR being above 1, *Pr*(OR > 1) or the Beta being above 0, *Pr*(Beta> 0)

Large *Pr*(OR > 1) or *Pr*(Beta> 0) values (e.g., > 0.95, or 0.99) indicate higher values for category 2 vs category 1 (see code of categorical variables). Conversely, small *Pr*(OR > 1) or *Pr*(Beta> 0) values (e.g., < 0.05, or 0.01), reflect higher category 1 vs category 2. Moreover, the probabilities *Pr*(OR > 1) or *Pr*(Beta> 0) can be interpreted as 1 - *Pr*(OR > 1) or as 1 – *Pr*(Beta< 0), respectively. Thus, probability values near 1 and 0 both indicate meaningful effects and are indicated in bold

Conditions A1 to A1 examined the influence of illness severity (in terms of frequency of relapse and intensity of symptoms)

Conditions B1 to B4 examined the influence of adding or not the name of illness in the clinical vignettes (comparing only the vignettes of illness with the highest degree of severity). Results of the uni- and multivariate analyses are not reported here and provided similar results as in Conditions A1 to A4

## Discussion

The aim of the present paper was to investigate whether and how people choose ICHA to treat their chronic illnesses, and to examine the factors driving two different patterns of ICHA use: in addition to or in place of conventional medicine. People hypothetically facing chronic illnesses selected ICHA as complementary treatment option in a large majority of cases, and only 4.8% (43/899) selected strictly conventional medicine (i.e., excluding ICHA) as unique treatment option. ICHA were mostly selected as secondary treatment option in addition to conventional medicine. The second most preferred treatment option placed ICHA before conventional medicine. All in all, the complementary attitude towards ICHA was largely predominant (41.1% of people selecting it as unique treatment option and 94.2%selecting this option at least once), whereas the alternative attitude was rarer (2.1% of people selecting it as unique treatment and 25.9% selecting this option at least once).

### Use of ICHA

Our results are in line with previous studies showing that more than 80% of patients with cancer use ICHA in addition to chemotherapy during the beginning of cancer treatment [27], indicating that patients facing severe illnesses often use ICHA as complementary treatment in the early phase of their illness. Previous national studies found that about 21.1 to 26.4% of people in the general population used ICHA at least once during the past 12 months [19, 28]. The prevalence was 19.7% in Germany, which is lower than what our results indicate. Moreover, previous studies indicated that gender, education or socio-economic level influence the use of ICHA [18, 19, 29]. In our study, neither gender nor education modulated the pattern of use of ICHA but we found that unemployed participants used ICHA as secondary treatment option (weak complementary) more often than other participants, who either preferred the alternative or the strictly conventional option. Those discrepancies might be explained by the particular design of our study that did not examine strictly the propensity to use ICHA in daily life, but people’s attitudes toward different pattern of use of ICHA when hypothetically facing chronic illness. It is worth mentioning that, people with poor health were more prone to use ICHA in the above cited national studies.

Our study revealed that the propensity to use ICHA is not a stable and fixed characteristic of individuals but varies according to the characteristic of the illness. In fact, 421 participants (46.8%) exhibited the same pattern of treatment preference whatever the descriptions of the clinical vignettes, while the other part of the sample (478 participants, 53.2%) adapted their choice and showed a flexible patterns of treatment preference. To our knowledge, this study is the first to demonstrate that variations exist in the propensity to consider ICHA as a valuable treatment option depending on the presentation of the illness to cure. Furthermore, among all treatment combinations, those including ICHA as complementary option (either after or before conventional treatments) were largely predominant (over 95%).

### Factors influencing the different patterns of use of ICHA

Our study design made it possible to examine the factors influencing the propensity to choose ICHA. The “strong complementary” pattern was preferred over the “weak complementary” pattern if the intensity of symptoms, the perceived social stigma and distress associated to the illness were low. This result appears to give credit to the label “soft medicines” considering that ICHA might be used as a first treatment option for trivial, non-severe health hassles. This interpretation should be nuanced by reminding that the illnesses presented in this study were all chronic, severe and potentially debilitating.

Surprisingly, the belief in the efficacy of treatment (being ICHA or conventional) did not influence the pattern of choice exhibited by the participants. This seems in contradiction with the literature showing that this factor importantly drove the propensity to choose ICHA (e.g., [19]. In fact, this might be explained by the fact that our analyses considered distinct patterns of use of ICHA and not only the issue of using ICHA or not. Complementary analyses restricted to the participants with single treatment option confirmed that belief in efficacy of ICHA clearly decreased and that of conventional medicine increased from “alternative” to “conventional” participants (data not shown). Altogether, the influence of the belief in efficacy of ICHA was observed in comparing participants with single pattern of choice, but this influence was not an obvious factor influencing the way people flexibly changed their attitudes towards ICHA.

Regarding the “alternative” pattern of choice, this option as single treatment option for all vignettes was marginal (9 participants, 2.1%). In contrast, combinations that included the “alternative” option represented 20.1 to 25.9% of all combinations, so that altogether, 133 (124 + 9, 14.8%) participants selected at least once the alternative option as a possible treatment option. This indicates that a substantial number of participants harbor both alternative and complementary attitudes towards ICHA depending on the situation they face. The preference for the alternative option was more frequent for illnesses perceived as less distressful and less treatable and to a lesser degree with low level of symptoms and low frequency of relapse. This is in line with the results mentioned above concerning the preference for the strong complementary option.

Finally, adding the name of the illness to the description of symptoms resulted in a preference for the weak over strong complementary option. Keeping in mind that the comparison was performed between the vignettes with the most severe clinical presentations (high frequency of relapse and high intensity of symptoms), this indicates that clarifying a diagnosis with patients alters their appreciation of the need for conventional over complementary medicines.

### Comparison between mental and somatic illnesses

Although the weak and strong complementary patterns were both selected in similar proportions in mental and somatic illnesses, mental illnesses were associated more frequently to “extreme” patterns of choice (namely alternative or strictly conventional). For instance, 65 participants (11.2%) compared to 49 (8.3%) selected the strictly conventional option as single treatment option for mental and somatic illnesses, respectively, and 30 participants (5.2%) compared to 15 (2.7%) selected the alternative option as single treatment option for mental or somatic illnesses, respectively. In total, 106 participants (30 + 76, 11.8%) selected at least once the alternative option for mental illnesses compared to 82 participants (15 + 67, 9.1%). Multivariate analyses confirmed that facing mental vs. somatic illnesses clearly influenced the switch from weak complementary to either alternative or strictly conventional treatment after controlling for other variables. Moreover, it is worth noting that our results did not show that complementary *medicines* were more preferred for mental vs. somatic illnesses but that alternative *attitudes* were more frequent for those illnesses. This result seems in line with the fact that about 60% of patients with mental disorder prefer using herbal remedies until they consider them not effective [6] and may reflect prominent negative attitudes toward psychotropic drugs [30, 31]. Further studies, however, are needed to tease out the reasons leading to these more extreme attitudes.

### Limitations

As for all web-based research, our sample was confined to people having access to the internet and included participants who were open to this kind of research. But since most people have internet access, potential biases might be higher in a clinical population in view of the large treatment gap in that most people with psychological disorders choose to remain untreated. This study investigated people who faced chronic illnesses hypothetically. Therapeutic preference might in fact differ between hypothetical and real-life situations as it might have been difficult for participants to fully understand/imagining the burden of having these diseases. Therefore, a mixed method design including both online and offline interviews with patients having these illnesses may have added to the validity of our study. While participation was comparable (16%) to previous online studies run with WiSoPanel [24], it does not allow generalizability of our findings. For all these reasons, our results must be interpreted with some caution. However, as we primarily aimed to target societal representations of ICHA, the present study provides a first and relevant indication about it. ICHA is a heterogeneous group of distinct medicines but was described quite globally in the present study. This is particularly true for the treatment options “alternative medicines” and “spirituality/religion” that were grouped together in our analyses as representatives of “complementary medicines”. Therefore, further questions remained unanswered: Which kind of ICHA is preferentially selected in case of a particular illness or of particular symptoms? How do patients express their motives or expectations associated with the use of ICHA?

## Conclusions

From the point of view of scientific medicine one may expect that people facing severe illnesses would mainly select conventional and evidenced-based treatments. This study provides new evidence that for both mental and somatic illnesses, medical pluralism [11, 12] is the norm, that is, the integration of complementary medicine in addition to conventional treatment, mostly as second line (but sometimes also as first line) treatment option. Bearing in mind the limitations due to the virtual nature of this study, our results highlight the need to take attitudes toward ICHA into account for a better understanding of patients’ preferences to use ICHA.

## Additional files

Additional file 1: Combinations of illness presentations. The table presents the different combinations of illness presentations (somatic vs. mental; with high vs. low frequency of relapse; with high vs. low symptoms severity) that participants were invited to read when starting the study. (DOCX 15 kb)

Additional file 2: Examples of coding of treatment preference. The document provides details on the method used to determine the different pattern of treatment preference depending on their selection of the proposed treatment option after each vignette. (DOCX 16 kb)

Additional file 3: JAGS script of the multivariate analysis. The following statistical script in R has been used to perform the multivariate statistical analysis (DOCX 15 kb)

## Abbreviations

ICHA: integrative and complementary health approaches
LAI: Long-acting injection
MS: Multiple sclerosis
RA: Rheumatoid arthritis
RD: Recurrent depression
SZ: Schizophrenia
