# Supplementary material for: Alternative or complementary attitudes toward alternative and complementary medicines
Source: BMC Complement Altern Med. 2019 Apr 8;19:83. doi: 10.1186/s12906-019-2490-z (PMC6454683; doi:10.1186/s12906-019-2490-z)
Supplement: Supplementary file 1 — Combinations of illness presentations. The table presents the different combinations of illness presentations (somatic vs. mental; with high vs. low frequency of relapse; with high vs. low symptoms severity) that participants were invited to read when starting the study. (DOCX 15 kb) [file 12906_2019_2490_MOESM1_ESM.docx]

**Additional file 1: Combinations of illness presentations.**

| Combination A1 | Combination A2 | Combination A3 | Combination A4 |
| --- | --- | --- | --- |
| Schizophrenia  RF: high  SE: high | Schizophrenia  RF: high  SE: high | Recurrent depression  RF: high  SE: high | Recurrent depression  RF: high  SE: high |
| Schizophrenia  RF: high  SE: low | Schizophrenia  RF: high  SE: low | Recurrent depression  RF: high  SE: low | Recurrent depression  RF: high  SE: low |
| Schizophrenia  RF: low  SE: high | Schizophrenia  RF: low  SE: high | Recurrent depression  RF: low  SE: high | Recurrent depression  RF: low  SE: high |
| Schizophrenia  RF: low  SE: low | Schizophrenia  RF: low  SE: low | Recurrent depression  RF: low  SE: low | Recurrent depression  RF: low  SE: low |
| Multiple sclerosis  RF: high  SE: high | Rheumatoid arthritis  RF: high  SE: high | Multiple sclerosis  RF: high  SE: high | Rheumatoid arthritis  RF: high  SE: high |
| Multiple sclerosis  RF: high  SE: low | Rheumatoid arthritis  RF: high  SE: low | Multiple sclerosis  RF: high  SE: low | Rheumatoid arthritis  RF: high  SE: low |
| Multiple sclerosis  RF: low  SE: high | Rheumatoid arthritis  RF: low  SE: high | Multiple sclerosis  RF: low  SE: high | Rheumatoid arthritis  RF: low  SE: high |
| Multiple sclerosis  RF: low  SE: low | Rheumatoid arthritis  RF: low  SE: low | Multiple sclerosis  RF: low  SE: low | Rheumatoid arthritis  RF: low  SE: low |
| Combination B1 | Combination B2 | Combination B3 | Combination B4 |
| Schizophrenia  RF: high  SE: high | Schizophrenia  RF: high  SE: high | Recurrent depression  RF: high  SE: high | Recurrent depression  RF: high  SE: high |
| Schizophrenia  RF: high  SE: low | Schizophrenia  RF: high  SE: low | Recurrent depression  RF: high  SE: low | Recurrent depression  RF: high  SE: low |
| Schizophrenia  RF: low  SE: high | Schizophrenia  RF: low  SE: high | Recurrent depression  RF: low  SE: high | Recurrent depression  RF: low  SE: high |
| Multiple sclerosis  RF: high  SE: high | Rheumatoid arthritis  RF: high  SE: high | Multiple sclerosis  RF: high  SE: high | Rheumatoid arthritis  RF: high  SE: high |
| Multiple sclerosis  RF: high  SE: low | Rheumatoid arthritis  RF: high  SE: low | Multiple sclerosis  RF: high  SE: low | Rheumatoid arthritis  RF: high  SE: low |
| Multiple sclerosis  RF: low  SE: high | Rheumatoid arthritis  RF: low  SE: high | Multiple sclerosis  RF: low  SE: high | Rheumatoid arthritis  RF: low  SE: high |
| Schizophrenia  with name of illness  RF: high  SE: high | Schizophrenia  with name of illness  RF: high  SE: high | Recurrent depression  with name of illness  RF: high  SE: high | Recurrent depression  with name of illness  RF: high  SE: high |
| Multiple sclerosis  with name of illness  RF: high  SE: high | Rheumatoid arthritis  with name of illness  RF: high  SE: high | Multiple sclerosis  with name of illness  RF: high  SE: high | Rheumatoid arthritis  with name of illness  RF: high  SE: high |
| Note: RF= relapse frequency, SE = severity of episodes | | | |
