# Supplementary material for: Alternative or complementary attitudes toward alternative and complementary medicines
Source: BMC Complement Altern Med. 2019 Apr 8;19:83. doi: 10.1186/s12906-019-2490-z (PMC6454683; doi:10.1186/s12906-019-2490-z)
Supplement: Supplementary file 2 — Examples of coding of treatment preference. The document provides details on the method used to determine the different pattern of treatment preference depending on their selection of the proposed treatment option after each vignette. (DOCX 16 kb) [file 12906_2019_2490_MOESM2_ESM.docx]

**Additional file 2: Examples of coding of treatment preference**

1. “Strictly conventional” for people selecting pill, LAI or psychotherapy as unique treatment option excluding ICHA or spiritual guidance
2. “Weak complementary” for people selecting pill or LAI as first treatment option combined with ICHA as further treatment option
3. “Strong complementary” for people selecting ICHA or spiritual guidance as first treatment option combined with conventional treatment as further treatment option
4. “Alternative” for people selecting ICHA or spiritual guidance as unique treatment option, excluding conventional treatment options (pill, LAI or psychotherapy)

Examples of treatment preference coded as Strictly conventional

| 1 | Pill | Pill | LAI | Pill |
| --- | --- | --- | --- | --- |
| 2 | LAI |  | Pill | Psychotherapy |
| 3 | Psychotherapy |  |  |  |
| 4 |  |  |  |  |
| 5 |  |  |  |  |
| 6 |  |  |  |  |

Examples of treatment preference coded as Weak complementary

| 1 | **Pill** | **LAI** | **LAI** | **Pill** |
| --- | --- | --- | --- | --- |
| 2 | ICHA | Psychotherapy | Pill | ICHA |
| 3 | Psychotherapy | ICHA | Spiritual guidance | Spiritual guidance |
| 4 | No treatment |  | ICHA | No treatment |
| 5 | Spiritual guidance |  | Psychotherapy | LAI |
| 6 |  |  | No treatment |  |

Examples of treatment preference coded as Strong complementary

| 1 | **ICHA** | **Spiritual guidance** | **ICHA** | **Spiritual guidance** |
| --- | --- | --- | --- | --- |
| 2 | Pill | LAI | Spiritual guidance | Psychotherapy |
| 3 | ICHA | Psychotherapy | Pill | ICHA |
| 4 | Psychotherapy | ICHA | Psychotherapy | No treatment |
| 5 | No treatment |  | No treatment | LAI |
| 6 |  |  | LAI |  |

Examples of treatment preference coded as Alternative

| 1 | ICHA | Spiritual guidance | ICHA | Spiritual guidance |
| --- | --- | --- | --- | --- |
| 2 | Spiritual guidance | ICHA | No treatment |  |
| 3 | No treatment |  |  |  |
| 4 |  |  |  |  |
| 5 |  |  |  |  |
| 6 |  |  |  |  |
