# Supplementary material for: Alternative or complementary attitudes toward alternative and complementary medicines
Source: BMC Complement Altern Med. 2019 Apr 8;19:83. doi: 10.1186/s12906-019-2490-z (PMC6454683; doi:10.1186/s12906-019-2490-z)
Supplement: Supplementary file 3 — JAGS script of the multivariate analysis. The following statistical script in R has been used to perform the multivariate statistical analysis (DOCX 15 kb) [file 12906_2019_2490_MOESM3_ESM.docx]

**Additional file 3: JAGS script of the multivariate analysis**

# transformation of the variable treatment preference into dummy variables with Weakcomplementary as reference category

# Coding of treatmentpref

# 1= Strictlyconventional

# 2= Weakcomplementary

# 3= Strongcomplementary

# 4= Alternative

Strictlyconventional<-ifelse(treatmentpref == 1, 1, 0)

Strongcomplementary<-ifelse(treatmentpref == 2, 1, 0)

Alternative<-ifelse(treatmentpref == 1, 1, 0)

# model of the multivariate model

model <-function(){

for (i in 1:N){

Strictlyconventional [i]~dbern(p_conv[i])

logit(p_conv[i])<- cons_conv + priorsuj[suj[i]]

+ b1_conv*age[i] #linear predictors

+ b2_conv*distress[i]

+ b3_conv*treatability[i]

+ b4_conv*socialstigma[i]

+ c1_conv*[frequency[i]] #categorical predictors

+ c2_conv*[intensityofsymptoms[i]]

+ c3_conv*[typeofillness[i]]

Strongcomplementary [i]~dbern(p_sc[i])

logit(p_sc[i])<- cons_sc + priorsuj[suj[i]]

+ b1_sc*age[i] #linear predictors

+ b2_sc*distress[i]

+ b3_sc*treatability[i]

+ b4_sc*socialstigma[i]

+ c1_sc*[frequency[i]] #categorical predictors

+ c2_sc*[intensityofsymptoms[i]]

+ c3_sc*[typeofillness[i]]

Alternative[i]~dbern(p_alt[i])

logit(p_alt[i])<-cons_alt + priorsuj[suj[i]]

+ b1_alt*age[i] #linear predictors

+ b2_alt*distress[i]

+ b3_alt*treatability[i]

+ b4_alt*socialstigma[i]

+ c1_alt*[frequency[i]] #categorical predictors

+ c2_alt*[intensityofsymptoms[i]]

+ c3_alt*[typeofillness[i]]

}

for(m in 1:NSuj) { #intra subject variance

priorsuj[m]~ dnorm(0,tau)

tau[m]~dgamma(0.1,0.1)

}

**#priors**

cons_conv~dnorm(0,0.01) #idem for cons_sc and cons_alt

b1_conv~dnorm(0,0.04) #idem for all linear predictors

c1_conv[1]<-0 #idem for all categorical predictors

c1_conv[1]~dnorm(0,0.04)
